# Dietary sodium estimation methods: accuracy and limitations of old and new methods in individuals at high cardiovascular risk

**DOI:** 10.1017/S1368980021004390

**Published:** 2022-04

**Authors:** Christiana Tsirimiagkou, Kalliopi Karatzi, Eirini D Basdeki, Antonios A Argyris, Theodore G Papaioannou, Maria Yannakoulia, Athanase D Protogerou

**Affiliations:** 1 Cardiovascular Prevention & Research Unit, Clinic & Laboratory of Pathophysiology, Department of Medicine, National and Kapodistrian University of Athens, 75, Mikras Asias Street, Athens 11527, Greece; 2 Department of Nutrition and Dietetics, School of Health Science and Education, Harokopio University of Athens, Greece; 3 Laboratory of Dietetics and Quality of Life, Department of Food Science & Human Nutrition, Agricultural University of Athens, Greece; 4 Hellenic Foundation for Cardiovascular Health and Nutrition, Athens, Greece; 5 Biomedical Engineering Unit, First Department of Cardiology, Department of Medicine, National and Kapodistrian University of Athens, Greece

**Keywords:** Dietary Na assessment, 24-h urine collection, Spot urine collection, 24-h dietary recall, FFQ

## Abstract

**Objective:**

Accurate and easy to use methods for dietary Na intake estimation in population level are lacking. We aimed at (i) estimating the mean Na intake in the group level using a variety of dietary methods (DM) and urinary methods (UM) and correlating them with 24-h urine collection (24UCol) and (ii) improving the accuracy of the existing DM.

**Design:**

The most common DM (three 24-h dietary recalls (24DR) and FFQ) and UM (24UCol and spot urine collection using common equations) were applied. To improve the existing: (i) 24DR, discretionary Na was quantified using salt-related questions or adding extra 15 % in total Na intake and (ii) FFQ, food items rich in Na and salt-related questions were added in the standard questionnaire (NaFFQ).

**Setting:**

National and Kapodistrian University of Athens, Greece.

**Participants:**

Totally, 122 high cardiovascular risk subjects (56·0 ± 12·6 years; 55·7 % males).

**Results:**

Mean 24 h Na excretion (24UNa) was 2810 ± 1304 mg/d. Spot urine methods overestimated the 24UNa (bias range: −1781 to −492 mg) and were moderately correlated to 24UCol (*r* = 0·469–0·596, *P* ≤ 0·01). DM underestimated the 24UNa (bias range: 877 to 1212 mg) and were weakly correlated with 24UCol. The improved DM underestimated the 24UNa (bias range: 877 to 923 mg). The NaFFQ presented the smallest bias (−290 ± 1336 mg) and the strongest correlation with 24UCol (*r* = 0·497, *P* ≤ 0·01), but wide limits of agreement in Bland–Altman plots (−2909 mg; 2329 mg), like all the other methods did.

**Conclusions:**

The existing methods exhibit poor accuracy. Further improvement of the newly developed NaFFQ could be promising for more accurate estimation of mean dietary Na intake in epidemiological studies. Additional validation studies are needed.

High Na intake is an important contributor to elevated blood pressure^(1)^, increasing CVD risk and mortality^(2,3)^. Although international organisations recommend a maximum daily Na intake of 2000 mg^(4)^, globally it is estimated to be almost double, reaching 3950 mg/d^(5)^. In large-scale epidemiological studies, the accurate estimation of dietary Na intake is important for detecting actual consumption and for identifying food items, food patterns or dietary behaviours related to Na intake and their association with diseases and treatments as well. In clinical settings also the assessment of Na intake is crucial for evaluating patients’ adherence to recommendations and guiding drug treatment decisions. A variety of urinary methods (UM) and dietary methods (DM) are available for the estimation of dietary Na intake; nevertheless, its accurate and precise quantification is still elusive^(6)^.

Spot urine samples, overnight urine collections and 24-h urine collections (24UCol) represent the UM. Based on the knowledge that about 90 % of Na consumed is excreted through urine during a 24-h period, the 24UCol is regarded as the gold-standard method^(7–9)^. However, it is a burdensome, time-consuming method and difficult to be applied in large-scale studies as well as in daily clinical practice of uncomplicated arterial hypertension management. Spot urine samples are more convenient to estimate 24-h urine Na excretion (24UNa) via specially designed equations^(10–16)^ (Table 1), which have been evaluated in several population groups^(17–19)^.

**Table 1:** Equations used to estimate 24-h urinary Na excretion from a single spot urine specimen

Kawasaki^(8)^	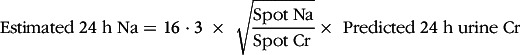 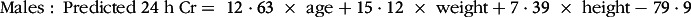 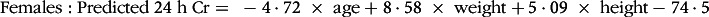	Estimated 24 h Na, mmol/d spot Na, mmol/l spot Cr: mg/l Predicted 24 h Cr: mg/d age, years weight, kg height, cm
Tanaka^(10)^	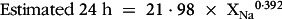 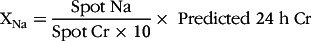 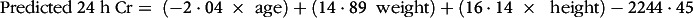	Estimated 24 h Na, mmol/d spot Na, mmol/l spot Cr: mg/dl Predicted 24 h Cr: mg/d age, years weight, kg height, cm
INTERSALT^(13)^	With Spot K 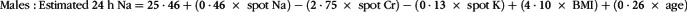 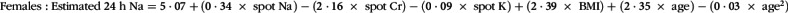 Without Spot K 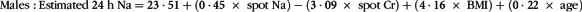 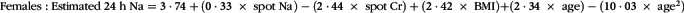	Estimated 24 h Na, mmol/d spot Na, mmol/l spot Cr: mmol/l spot K: mmol/l BMI: kg/m^2^ age, years
Toft^(11)^	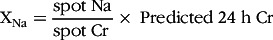 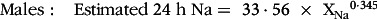 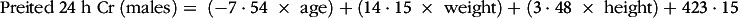	Estimated 24 h Na, mmol/d spot Na, mmol/l spot Cr: mg/dl Predicted 24 h Cr: mg/d age, years weight, kg height, cm
	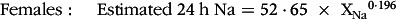 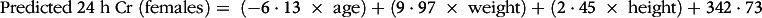	
Mage^(9)^	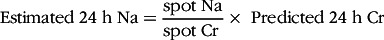 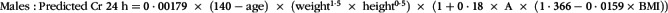 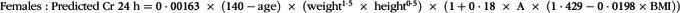 A = African American or Black race = 1/other race = 0	Estimated 24 h Na, mmol/d spot Na, mmol/l spot Cr: mg/dl Predicted 24 h Cr: mg/d BMI: kg/m^2^ weight, kg height, cm

On the other hand, the most common DM for Na estimation include 24-h dietary recalls (24DR), FFQ and diet records. These methods are commonly used in population-based studies, as they are efficient to highlight food items rich in Na; however, numerous methodological disadvantages exist^(6)^. A major one is the inability of all these methods to quantify the discretionary use of salt (table salt or use of salt during cooking), which has been previously reported to contribute significantly to the total Na intake^(20–22)^.

Several efforts have been made to develop an optimal diet-based tool for the estimation of mean Na intake on group level, with the majority of them focusing on short FFQ^(23–26)^, which are brief, easily completed and estimate Na intake through larger time periods compared with other DM. 24-h dietary recalls and food records are also suitable to cover a longer time periods if they are repeated. Nevertheless, usually FFQ are developed for particular population groups and designed according to their culture, dietary habits and traditional recipes, thus they may not be accurately applied to other populations.

To our knowledge, studies evaluating simultaneously the accuracy of different UM and DM for Na estimation with the gold-standard 24UCol are scarce. Moreover, there are no accurate DM for the quantification of discretionary salt, designed specifically for high CVD risk populations, for whom the identification of Na intake is essential. Taking into consideration all these issues, the aim of the present study is to (a) estimate the mean Na intake of population using a variety of DM and UM; (b) correlate these methods with the gold-standard 24UCol and (c) improve the existing DM in order to be more accurate in estimating the mean Na intake in population level.

## Methods

### Study design and population

A cross-sectional study was performed from January 2017 until October 2018. The study population consisted of consecutive and consenting to participate individuals at high CVD risk due to the presence of CVD risk factors (suspected or established treated or untreated hypertension, dyslipidaemia, diabetes mellitus and/or chronic inflammatory diseases). In order to detect a minimum difference of 500 mg in daily Na intake between each Na estimation method and the 24UNa (*α* = 0·05, power = 0·80), the minimum sample size for each pair of methods was calculated (*n* 60)^(27,28)^. To account for attrition (non-participation, missing data or incomplete 24UCol), which was estimated to be 50 %, 120 individuals were invited to participate. The study was approved by the ethical/scientific committee. All participants provided informed consent and underwent dietary and urinary assessment simultaneously, which was completed within 1 month.

#### Assessment of dietary sodium intake using urinary methods

##### Twenty-four hour urine collection

Participants were asked to keep one 24UCol following written and verbal instructions and a standardised protocol. The instructions were to carry out the collections from Sunday awakening and for the next 24 h, discarding the first morning void without: (a) missing voids and (b) any changes in their diet or medicine (the past 1 month). To verify completeness, sensitivity analyses were conducted after applying all available criteria for 24-h urine completeness^(10,29–31)^ (Statistical analysis section). Na derived from the 24UCol was calculated using the following equation:

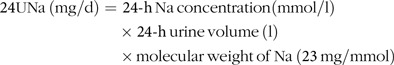




##### Spot urine

Participants were also asked to keep a single spot urine sample of the first morning void in proper bottle. In order to estimate the 24-h Na excretion from spot urine specimens, the most common conversion equations were applied^(11–13,16)^ (Table 1).

#### Assessment of dietary sodium intake using existing dietary methods

##### Twenty-four hour dietary recalls

Three 24DR using multiple-pass method were conducted (2 weekdays and 1 weekend day with a 7-d interval) by well-trained dietitians via telephone or face-to-face interviews. Participants were asked to report all the foods and beverages they consumed and their quantities the previous 24 h. With the use of a relevant nutrient analysis software (Nutritionist Pro, version 5.2, Axxya Systems-Nutritionist Pro, Stafford, TX, USA), food data from the 24DR were analysed in terms of macronutrient and micronutrient intake. The average of Na intake of the 3 d was used. If less than three 24DR were available, the average of the rest was used.

##### Food frequency questionnaire

In the first week of the dietary assessment, all participants were asked to complete a semi-quantitative FFQ, which is repeatable and valid for nutritional assessment regarding energy and macronutrients^(32)^. The FFQ consisted of a list of sixty-nine main food groups (i.e. cereals and starchy foods, fruits, vegetables, dairy products, meat, fish, legumes, added fats, sweets and alcoholic beverages) as well as questions related to dietary behaviours and habits^(32)^. Participants were asked to report the frequency of the consumption of these food groups the last month on a six-grade scale (from never/rarely to more than 2 times/d) in pre-specified amounts of food expressed in grams, ml or other common measures^(33)^. More details for FFQ development have been previously described^(32,34)^.

Daily food consumption was calculated as

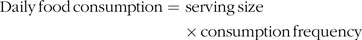




where consumption frequency was: never = 0; 1–3 times/month = 0·07; 1–2 times/week = 0·21; 3–6 times/week = 0·64; 1 time/d = 1; ≥ 2 times/d = 2.

The Na estimation for each food group was calculated as

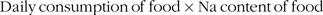




derived from United States Department of Agriculture (USDA) and local food composition tables^(35–37)^.

#### Assessment of dietary sodium intake using improved dietary methods

##### 24DR plus discretionary salt questions

In order to estimate discretionary salt, participants were asked to answer two salt-related questions separately for breakfast, lunch and dinner for each one of the 24DR:

- Question 1: How much salt did you use during the preparation of your meal?
  a = none, b = a little, c = moderate, d = a lot
- Question 2: Did you add extra salt on your plate (table salt)?
  a = no, b = yes.

For question 1, the following Na quantities were applied for each answer: a = none = 0 mg of Na, b = a little = 50 mg of Na per 100 g of food, c = moderate = 350 mg of Na per 100 g of food, d = a lot = 600 mg of Na per 100 g of food. These estimations were based on relevant statements/assessments from the Hellenic Food Authority (EFET)^(38)^: *‘If a food contains more than 0·6 g of sodium (or 1·5 g of salt) per 100 g, then it is high in sodium/ salt. If a food contains 0·1 g of sodium or less per 100 g then it is low in sodium/salt. If the amount of salt per 100 g is between these values, then the food contains a medium level of salt’.* Portion sizes from the 24DR were calculated in grams based on food equivalents and local food composition tables^(36)^.

For question 2, the answer ‘yes’ was defined as 2 dashes of salt, which are equivalent to 775 mg of Na^(35)^ and when the answer was ‘no’, no Na (0 mg) was added. The mean Na derived from questions 1 and 2 was then added to the Na derived from the 24DR and was calculated as

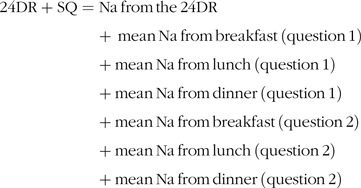




The Na of the meals (breakfast, lunch and dinner) was calculated based on the estimations of Na intake from questions 1 and 2 (average from the three 24DR).

##### 24DR plus 15 %

An alternative way to estimate discretionary use of salt was applied. We calculated the discretionary Na based on the assumption that Na from cooking and table is 15 % of the total Na intake for our population, as previously reported^(20–22)^. In specific, total Na intake was then calculated as

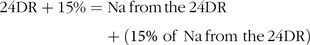




##### Sodium FFQ

In order to improve Na estimation, the food list of the previously mentioned FFQ was extended with food items rich in Na and questions regarding dietary behaviours related to discretionary use of salt (NaFFQ). The added food groups and questions are presented in the Supplement (see online Supplemental Table 1). The foods items added were salted butter and margarine, several rich in Na cheeses (e.g. roquefort, parmesan, edam, gouda, gruyere, etc.), salty crackers/biscuits, canned fish/seafood and refined tomato juice. To estimate Na added in cooked meals and salads, Question b of the NaFFQ (*How much salt do you use in your cooked meals and salads?* see online Supplemental Table 1) was used, according to Hellenic Food Authority (EFET)^(38)^ as mentioned above. Participants’ answers were calculated as none = 0 mg Na, a little = 50 mg Na/100 g of food, moderate amount = 350 mg Na/100 g of food, much = 600 mg Na/100 g of food, very much = 900 mg Na/100 g of food. Then the quantified Na derived from participants’ response in Question b was added to each cooked meal and salad per 100 g of food of the NaFFQ. Na was then calculated as

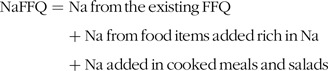




Cooked meals included rice, potatoes, red and white meat, fish & seafood, legumes, traditional dishes and home-made pies. Salads included all vegetables, raw or boiled.

### Assessment of anthropometric parameters

Participants’ weight was measured without shoes or heavy clothes to the nearest 0·1 kg (Tanita Body Composition Analyzer, BC-418). Height was measured without shoes, with the participants standing with their shoulders relaxed, their arms hanging freely and their head in Frankfurt horizontal plane (SECA 213). BMI was calculated as weight/(height)^2^ (kg/m^2^).

### Assessment and definition of CVD risk factors

Hypertension was defined as the use of antihypertensive drugs and/or office blood pressure measurement >139/89 mmHg (average of three sequential readings with 1-min interval in the supine position after at least 10 min of rest; Microlife WatchBP Office, Microlife AG, Widnau, Switzerland)^(39)^. Dyslipidaemia was defined as the use of lipid-lowering drugs and/or LDL-cholesterol level >160 mg/dl. Diabetes mellitus was defined as fasting glucose higher than 126 mg/dl or HbA1c ≥6·5 % and/or glucose-lowering treatment. Smoking or vaping was defined by the use of at least one cigarette/d each day of the week or the use of e-cigarette.

### Statistical analysis

All the analyses were conducted using SPSS version 25 (IBM Corp. Released 2017, IBM Corp.). Continuous variables are presented as mean ± sd and categorical variables as absolute frequency and percentage (%). Significance levels were set at *P*-value < 0·05. Distribution normality of the variables was tested using the Kolmogorov–Smirnov test and histograms. The differences between methods (bias of mean values) were calculated as 24UNa minus the Na measures of the other DM and UM. Paired samples *t*-test and Wilcoxon test, when appropriate, were used to determine the significance of differences of mean values of Na. To assess the correlation between 24UCol and the other Na estimation methods, the Pearson’s correlation coefficient (for normally distributed variables) and Spearman correlation coefficient (for variables not normally distributed) were applied. Consistency between different methods of Na estimation was also assessed with the intraclass correlation coefficient (ICC)^(40)^. It is generally accepted that there is no absolute interpretation of ICC values. However, in the present study, we used the recommendation of Koo and Li^(41)^; accordingly, ICC values <0·5 are indicative of poor reliability, ICC between 0·5 and 0·75 indicate moderate reliability, ICC between 0·75 and 0·9 indicate good reliability, and ICC values greater than 0·90 indicate excellent reliability.

Bland–Altman plots were used to evaluate differences between Na estimation methods and the 24UCol and evaluate the agreement between them^(42,43)^. The upper and lower limits of agreement between two different estimates of Na were calculated by the mean difference ± 1·96 × sd of differences. Linear regression analysis was used to evaluate associations in difference and mean (between 24UCol and each Na estimation method). The analyses regarding correlations between 24UCol and each Na estimation method and ICC as well were repeated after excluding all subjects having incomplete 24UCol (sensitivity analysis) and they are presented in the supplement. The exclusion criteria for incomplete 24UCol were set according to international bibliography^(10,29–31)^ and are presented in the supplemental material (see online Supplemental Table 2).

## Results

One hundred and twenty-two (122) participants with available 24UCol data were used for the analyses (56·0 ± 12·6 years; 55·7 % males) (Table 2). The available sample size for UM and DM was Spot UM, *n* 71; 24DR = 119; FFQ, *n* 87; NaFFQ, *n* 60 (Table 2). Descriptive characteristics of study population are presented in Table 2. Incomplete collections presented the 7·4 % of participants (Table 2).

**Table 2:** Descriptive characteristics of the study population for the total sample and each Na estimation method

	Urinary methods						Dietary methods							
	24UCol *n* 122		Spot urine *n* 71				24DR *n* 119				FFQ *n* 87		NaFFQ *n* 60	
	Mean	sd	Mean	sd			Mean	sd			Mean	sd	Mean	sd
Age, years	56·0	12·6	56·2	11·9			55·9	12·6			56·1	13·1	56·4	12·3
Weight, kg	80·7	18·0	81·4	18·9			80·4	18·1			80·3	18·7	80·1	18·9
Height, cm	169·9	11·3	170·7	11·3			169·9	11·4			170·3	11·6	170·8	11·9
BMI, kg/m^2^	27·9	5·6	27·9	6·0			27·9	5·6			27·6	5·2	27·3	5·1
Energy, kcal/d	–	–	–	–			1998·8	668·3			2238·7	713·7	2210·7	695·5
					*Existing 24DR*		*24DR + 15 %*		*24DR + SQ*					
Na derived from food, mg/d	–	–	–	–	1633·8	763·6	1633·8	763·6	1633·8	763·6	1704·3	800·0	1793·5	873·5
Na derived from table salt, mg/d	–	–	–	–	–	–	288·3	134·8*	58·6	90·3	–	–	1197·4	1047·2*
Na derived from cooking salt, mg/da	–	–	–	–	–	–			276·4	261·6	–	–		
	(%)		(%)				(%)				(%)		(%)	
Males	55·7		56·3				55·5				57·5		60·0	
Smoking														
Current (cigarette/e-cigarette)	40·5		43·6				41·5				40·7		38·8	
Ex smoking	20·7		22·5				20·3				19·8		23·3	
Never	38·8		33·8				38·1				39·5		38·3	
CVD	10·7		7·1				11·0				11·5		10·0	
T1DM	1·6		1·4				1·7				2·3		1·7	
T2DM	8·2		11·3				8·4				8·0		10·0	
DMS drugs	5·7		7·0				5·9				6·9		6·7	
Hypertension	64·8		60·6				63·9				65·5		58·3	
Hypertension drugs	46·7		42·3				45·4				48·3		43·3	
Dyslipidaemia	65·6		64·8				65·5				66·7		68·3	
Dyslipidaemia drugs	33·6		32·4				33·6				31·0		25·0	
Autoimmune/inflammatory disease	13·2		15·5				12·7				11·5		13·3	
Infectious disease	30·6		32·4				30·5				29·9		31·7	
Incomplete 24 h UCol	7·4													

24UCol, 24-h urine collection; 24DR, 24-h dietary recalls (three 24DR were performed); 24 DR + 15 %, 24-h dietary recalls Na plus 15 % (discretionary Na); 24 DR + SQ, 24-h dietary recalls Na plus discretionary salt questions; T1DM, type 1 diabetes mellitus; T2DM, type 2 diabetes mellitus.

* Na derived from table and cooking salt.

Table 3 presents mean Na intake or excretion for all the available UM and DM applied, as well as the significance of the differences between 24UNa and each one of the other Na estimation methods. Mean 24UNa was 2810·4 ±1303·9 mg/d. Regarding spot urine methods, all of them overestimated 24UNa (mean bias range: −1780·9 to −492·0 mg) with the INTERSALT without spot K equation presenting the smallest bias (−492·0 ± 1223·2 mg) (Table 4). Regarding the existing DM, both of them underestimated 24UNa (mean bias range: 876·6 to 1211·6 mg). From the improved DM, 24DR + 15 % and 24DR + SQ underestimated 24UNa (876·6 ± 1342·6 and 923·3 ±1345·8 mg, respectively, *P* < 0·001), but the NaFFQ marginally overestimated 24UNa showing the smallest bias from all DM and UM (−290·2 ± 1336·2 mg) (Table 3).

**Table 3:** Na intake/excretion for each dietary and urinary Na estimation method, bias of mean values and comparisons with the 24-h urine collection

	*n*	Na intake or excretion mean	SD	Bias (24UNa minus each Na estimation method) mean	SD	*P*
Urinary methods						
24UCol, mg/d	122	2810·4	1303·9	–		–
Kawasaki, mg/d	71	4523·0	1331·0	−1780·9	1235·2	<0·001
Tanaka, mg/d	71	4862·1	10 633·2	−894·8	1154·1	<0·001
INTERSALT with spot K, mg/d	67	3209·4	869·0	−599·0	1140·0	<0·001
INTERSALT without spot K, mg/d	71	3207·8	843·1	−492·0	1223·2	0·001
Mage, mg/d	71	3438·8	2494·8	−722·6	2050·6	0·016
Toft, mg/d	71	3852·8	955·7	−1136·6	1165·6	<0·001
Dietary methods						
Existing dietary methods						
24 DR, mg/d	119	1633·8	763·6	1211·6	1298·8	<0·001
FFQ, mg/d	87	1704·3	800·0	1058·7	1335·7	<0·001
Improved dietary methods						
24 DR + 15 %, mg/d	119	1922·2	898·3	923·3	1345·8	<0·001
24 DR + SQ, mg/d	119	1968·9	917·0	876·6	1342·6	<0·001
NaFFQ, mg/d	60	2990·9	1397·5	−290·2	1336·2	0·098

24UCol, 24-h urine collection; 24UNa, 24-h urine Na; 24 DRNa, 24-h dietary recalls Na; 24 DR + 15 %, 24-h dietary recalls Na plus 15 % (discretionary Na); 24 DR + SQ, 24-h dietary recalls Na plus discretionary salt questions.

**Table 4:** Pearson’s and spearman correlations & intraclass correlation coefficients between 24-h urine collection and the other Na estimation methods

		Na estimation methods		Total sample	95 % CI
Spot urine methods		Kawasaki	*r*	0·583**	
			ICC	0·74	0·58, 0·84
			*n*	71	
		Tanaka	*r*	0·542**	
			ICC	0·66	0·46, 0·79
			*n*	71	
		INTERSALT with spot K	*r*	0·492**	
			ICC	0·63	0·40, 0·77
			*n*	67	
		INTERSALT without spot K	*r*	0·469**	
			ICC	0·59	0·35, 0·75
			*n*	71	
		Mage	*r*	0·596**	
			ICC	0·65	0·44, 0·78
			*n*	71	
		Toft	*r*	0·570**	
			ICC	0·68	0·48, 0·80
			*n*	71	
Dietary methods	Existing dietary methods	24DR	*r*	0·263**	
			ICC	0·40	0·14, 0·58
			*n*	119	
		FFQ	*r*	0·232*	
			ICC	0·39	0·06, 0·60
			*n*	87	
	Improved dietary methods	24DR + SQ	*r*	0·296**	
			ICC	0·44	0·20, 0·61
			*n*	119	
		24DR + 15 %	*r*	0·263**	
			ICC	0·42	0·17, 0·60
			*n*	119	
		NaFFQ	*r*	0·497**	
			ICC	0·66	0·43, 0·80
			*n*	60	

ICC, intraclass correlation coefficient; 24UNa, 24-h urine Na; 24 DRNa, 24-h dietary recalls Na; 24DRNa + 15 %, 24-h dietary recalls Na plus 15 % (discretionary Na); 24-h DRNa + SQ, 24-h dietary recalls Na plus discretionary salt questions.

* *P* < 0·05.

** *P* ≤ 0·01.

Table 4 presents Pearson’s and Spearman’s correlation tests as well as the ICC between 24UCol and each one of the UM and DM. Regarding spot urine methods, Mage equation exhibited the strongest correlation with 24UCol (*r* = 0·596, *P* < 0·001), and all other equations presented moderate reliability (ICCs range: 0·59–0·74). From the existing DM, both of them weakly correlated to 24UCol (*r* = 0·232–0·263, *P* < 0·05). Regarding the improved DM, 24DR + 15 % and 24DR + SQ were weakly correlated to 24UCol (*r* = 0·263-0·296, *P* ≤ 0·01) and presented poor reliability (ICC range: 0·42–0·44), but NaFFQ exhibited the strongest correlation with 24UCol (*r* = 0·497, *P* ≤ 0·01) and was moderately reliable (ICC 0·66 (95 % CI 0·43, 0·80)). In subgroup analysis (data presented in the Supplement – see online Supplemental Table 3): (a) four out of the five subgroups agreed that Mage equation exhibited the strongest correlation with the 24UCol (*r* = 0·625–0·700, *P* < 0·001) and (b) three out of the five agreed that Kawasaki equation was the only method presenting good reliability (ICC range: 0·76–0·80) and all the five subgroups agreed that regarding the existing and the improved DM, NaFFQ was the only method presenting moderate reliability (ICCs range: 0·44–0·51), while all the other DM presented poor reliability (ICC range: 0·20–0·32).

**Fig. 1:**
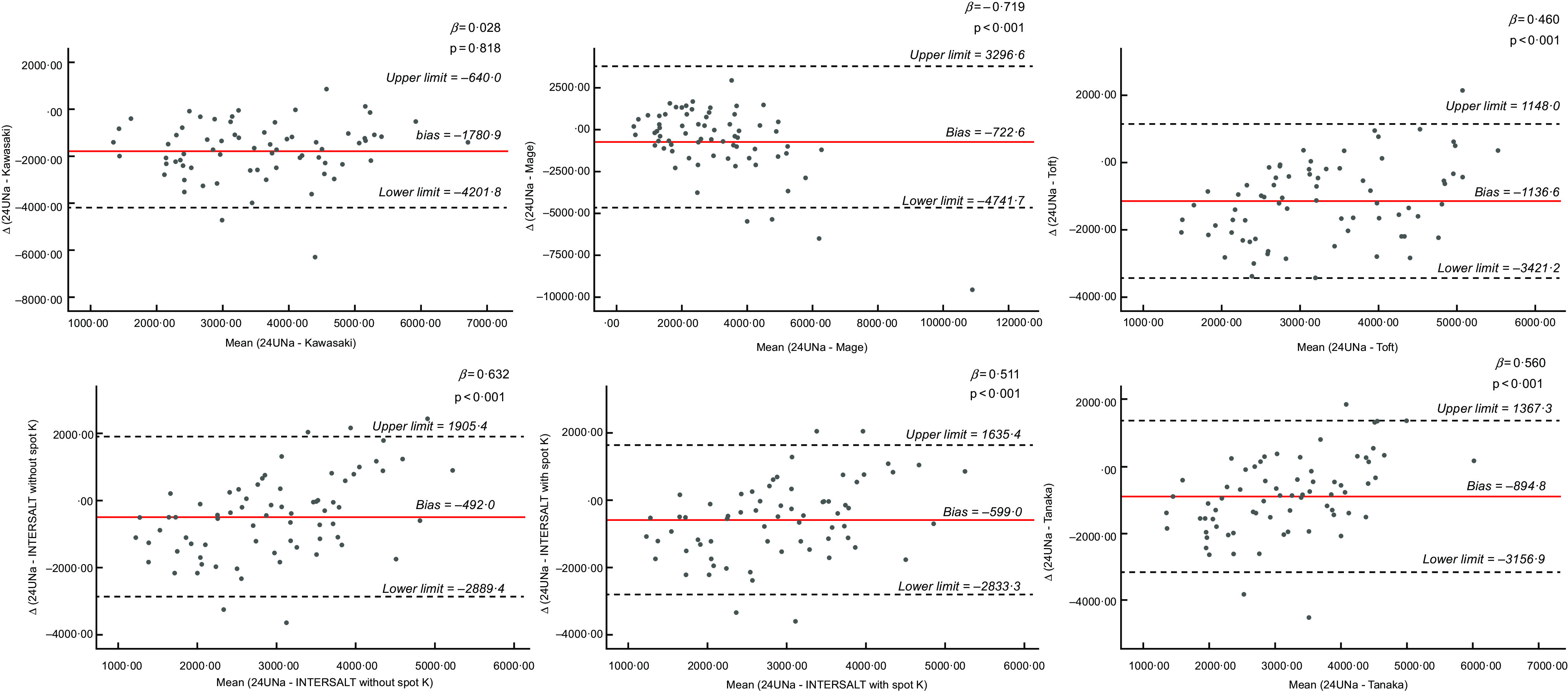
Bland–Altman plots comparing 24-h urinary Na excretion with Na estimated by spot urine equations. Solid line is the mean difference between methods and dashed lines are the 95 % CI of the difference between methods. Limits of agreement of the two Na assessment methods, defined as mean difference ± 1·96 × sd of differences. 24UNa, Na estimated by 24-h urine collection

**Fig. 2:**
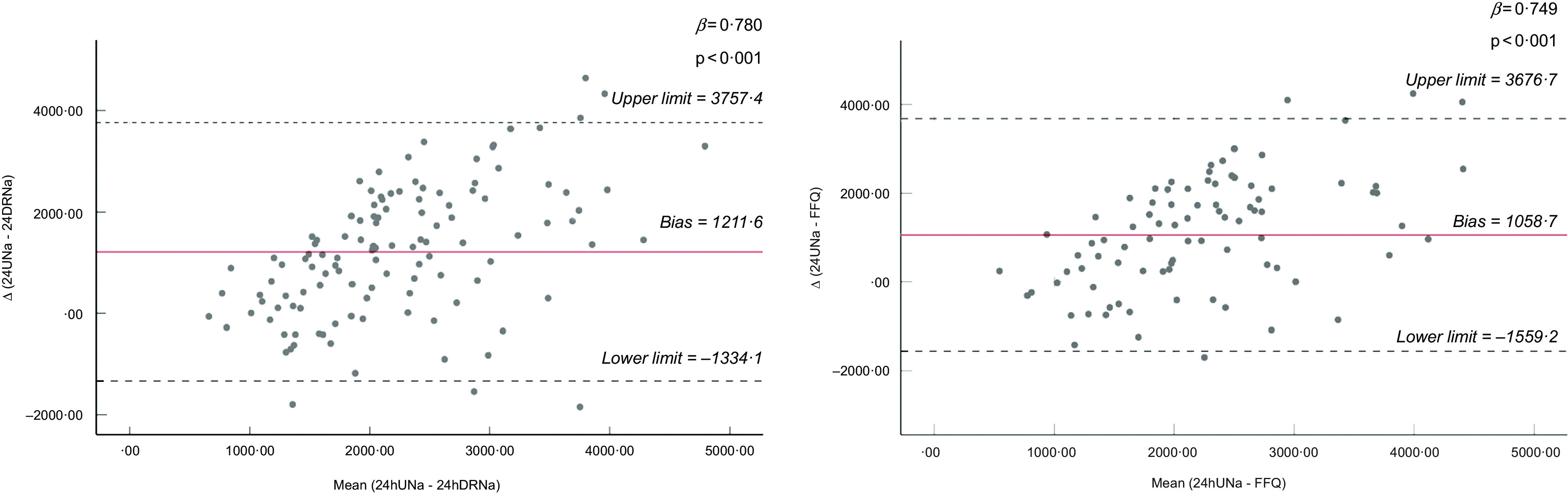
Bland–Altman plots comparing 24-h urinary Na excretion with Na estimated by existing DM. Solid line is the mean difference between methods, and dashed lines are the 95 % CI of the difference between methods. Limits of agreement of the two Na assessment methods, defined as mean difference ± 1·96 × sd of differences. 24UNa, Na estimated by 24-h urine collection; 24-h DRNa, Na estimated by 24-h dietary recalls

**Fig. 3:**
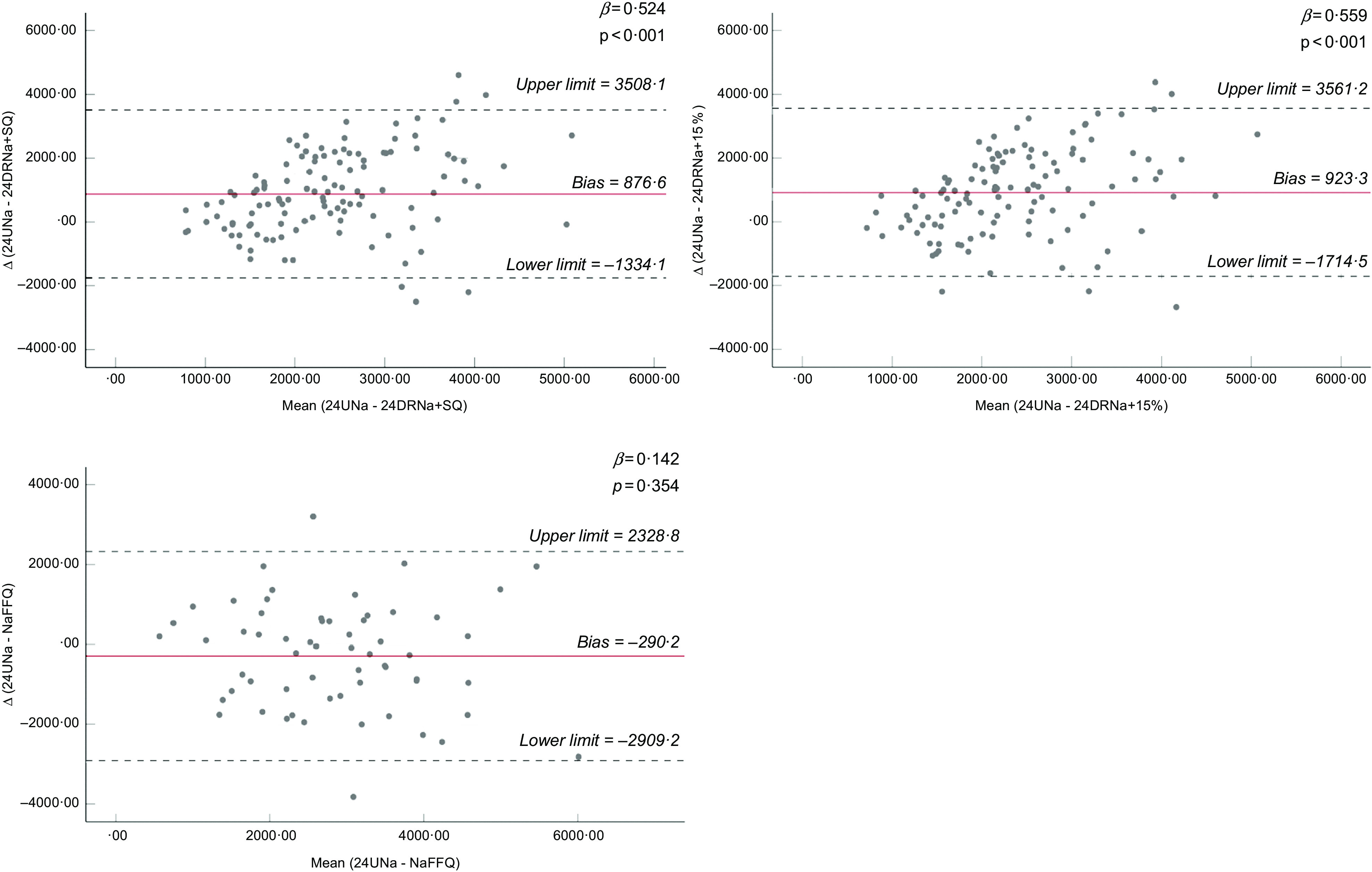
Bland–Altman plots comparing 24-h urinary Na excretion with Na estimated by improved DM. Solid line is the mean difference between methods and dashed lines are the 95 % CI of the difference between methods. Limits of agreement of the two Na assessment methods, defined as mean difference ± 1·96 × sd of differences. 24UNa, sodium estimated by 24-h urine collection; 24-h DRNa + SQ, Na estimated by 24-h dietary recalls plus salt-related questions

Bland–Altman plots for all the spot urine methods, existing DM, and improved DM are presented in Figs 1, 2, and 3, respectively. Regarding spot urine methods, the use of equations of Toft, INTERSALT with spot K and INTERSALT without spot K resulted in underestimation at lower levels and overestimation at higher levels of Na excretion in Bland–Altman plots (Fig. 1). On the contrary, Mage equation was the only method providing the opposite finding, presenting overestimation at low levels of Na excretion and underestimation at higher levels. Finally, the Kawasaki equation exhibited a homogeneous variation as Na excretion levels increase (Fig. 1). All methods presented wide ranges of agreement (Kawasaki: −4201·8 to 640·0; Mage: −4741·7 to 3296·6; Toft: −3421·2 to 1148·0; INTERSALT without spot K: −2889·4 to 1905·4; INTERSALT with spot K: −2833·3 to 1635·4; Tanaka: −3156·9 to 1367·3) (Fig. 1). Linear regression analysis revealed statistically significant associations between the difference and the mean of 24UCol and all the spot urine methods, except from the Kawasaki equation (*β* = 0·028, *P* = 0·818) (Fig. 1). Regarding the existing DM (Fig. 2), both of them presented consistent bias in Bland–Altman plots, underestimating the 24UNa in low levels of Na intake and overestimating in high levels of Na intake, while presenting wide ranges of agreement in Bland–Altman plots (24DR: −1334·1 to 3757·4; FFQ: −1559·2 to 3676·7) (Fig. 2). Linear regression analysis revealed statistically significant association between the difference and the mean of 24UCol and all the DM (Fig. 2). Regarding the improved DM, the NaFFQ was the only one showing: (a) a homogeneous variation as the mean Na intake increases in Bland–Altman plots, however, presenting wide ranges of agreement (−2909·2 to 2328·8) and (b) not statistically significant association between the difference and the mean of 24UCol and improved DM in linear regression analysis (*β* = 0·142, *P* = 0·354) (Fig. 3). The other two improved DM (24DR + 15 % & 24DR + SQ) underestimated the 24UNa at low levels of Na intake and overestimated at high levels of Na intake, presenting wide ranges of agreement (24DR + SQ: −1334·1 to 3508·1; 24DR + 15 %: −1714·5 to 3561·2) (Fig. 3).

All the analyses were repeated using 1 dash of salt instead of 2 in the *question 2* of the improved dietary recalls (24DR + SQ), and similar findings were observed (data not shown).

## Discussion

This study aimed to assess and compare the most commonly used in population studies UM and DM for mean Na intake and develop a new accurate and easy to use clinical tool for Na estimation in high CVD risk populations. The main findings of this study are (i) the existing DM tend to underestimate and spot urine methods tend to overestimate the true Na intake; (ii) all the existing DM are weakly correlated and present poor agreement with the 24UCol, and all the spot urine methods are moderately correlated and present moderate agreement with the 24UCol and (iii) the new NaFFQ is the only method that performed better in the analysis, having simultaneously the smallest bias in mean differences, the strongest correlation with the 24UCol regarding DM and a homogeneous variation as the mean Na intake increases in Bland–Altman plots, but still wide limits of agreement.

Spot urine collection is an easily applicable alternative in estimating dietary Na intake. Increasing studies aim to reveal the most accurate formula for converting spot Na to 24UNa, comparing not only those commonly used^(17,44–48)^ but also those newly designed^(49,50)^ against the gold-standard 24UCol. The mostly studied formulas are the INTERSALT equation, the Tanaka equation and the Kawasaki equation. Despite some controversies^(50)^, a large number of studies support that among the existing equations, the INTERSALT performs better in estimating the 24UNa showing the least bias^(44,45,49,51,52)^. In our findings, the INTERSALT equation presented the lowest bias among all the other equations; however, it was moderately correlated with 24UNa and also presented consistent bias in Bland–Altman plots by underestimating Na intake at low levels of Na excretion and overestimating at high levels of Na excretion. However, it is important to note that the studies supporting the use of INTERSALT equation as the best alternative of 24UCol for Na estimation have all been conducted in general populations^(44,45,49,51,52)^, which is in contrast to our high CVD risk population. Indeed, the evidence is not supportive of the use of the INTERSALT equation in high-risk patients, having chronic diseases such as chronic kidney disease or hypertension^(46,53)^. Dougher *et al*. compared commonly used equations for Na estimation in 129 chronic kidney disease patients^(53)^. According to their findings, the authors conclude that spot urine equations do not estimate accurately dietary Na intake in this group of people. Similarly, when Ma *et al.* assessed Na intake by the INTERSALT, the Tanaka and the Kawasaki equations in 365 high-risk stroke patients, they found poor correlations (*r* = 0·35–0·38), poor reliability (ICCs = 0·31–0·38) and significant biases among all the three methods compared with the 24UCol^(46)^. These findings are in agreement not only with our study but also with a significant number of studies, which do not recommend the use of spot equations for dietary Na estimation^(17,47,48,54,55)^. It is important to note that Na excretion presents a circadian variability, which potentially could influence the estimations derived from spot urine collections. A systematic review of studies comparing the 24UCol and spot urine collections for estimating salt intake, conducted by Ji *et al.*, included twenty studies and 1·380·130 participants, concluded that although it is of great interest to replace the 24UCol as a method for Na intake estimation, the best alternative UM remains uncertain as a wide range of correlations (*r* = 0·17–0·94) between 24UCol and the other methods presented in their work^(56)^. Also, a systematic review and meta-analysis in 10·414 participants from thirty-four countries showed that *‘estimates based upon spot urine samples have excellent sensitivity (97 %) and specificity (100 %) at classifying mean population salt intake above or below the World Health Organization maximum target of 5 g/d but underestimate intake at high levels of consumption and overestimate at lower levels of consumption’* ^(57)^. Even more interestingly, in a recent analysis of TOHP (Trials of Hypertension Prevention) study follow-up data, conducted by He *et al*., estimated values of Na excretion (using the Kawasaki, INTERSALT with spot K and Tanaka equations) – examining the same population sample – altered the linear association between 24UNa and mortality to J- or U-shaped^(58)^. The authors concluded that these urinary Na estimation methods *‘were systematically biased with overestimation at lower levels and underestimation at higher levels’*, indicating that estimation of Na through spot urine specimens is inaccurate^(58)^. All these findings are consistent to a WHO/PAHO statement in the protocol for population level Na determination in 24-h urine sample, declaring that *‘the use of spot-urine is discouraged as a method to determine Na, potassium or iodine intake because of the limitations and uncertainty inherent in the method’*.

As regard to the existing DM for Na estimation, although it is useful and efficient to highlight food items rich in Na, several methodological disadvantages have been raised. The most commonly discussed include the difficulty or even inability to assess and quantify discretionary Na; deviated estimations of Na due to high variability in Na content in recipes of homemade and manufactured food; the absence of Na derived from medicines and dietary supplements and participant-related issues (underreporting and difficulty to recall all the food and beverages consumed; socially desired answers and dietary behaviour modification)^(6)^. A small number of studies suggest that DM, such as food diaries or multiple 24DRs, can be used for Na estimation, having the ability to predict over 90 % of 24UNa^(20,59)^. However, the majority of the available studies have reported that Na estimation based on DM tends to underestimate 24UNa (levels of underreporting 15–40 %) and correlates weakly or moderately with 24UNa (*r*≈0·15–0·50)^(20,60–65)^. This is in line with our findings, showing weak correlations, poor reliability and high levels of bias, suggesting that the existing DM for Na estimation are inaccurate. In a recent meta-analysis including twenty-eight studies, McLean *et al.* compared 24DR with 24UCol^(66)^. 24DR underestimated mean Na intake by 607 mg/d, but high quality 24DR improved accuracy. The authors concluded that 24UCol remains the most accurate method to assess population Na intake; however, high-quality 24DR (use of multiple pass methods, accurate food composition databases and quantification of discretionary salt) could be used if 24UCol is not feasible^(66)^.

To our knowledge, studies comparing different DM and UM simultaneously for Na intake estimation are scarce. A recent study compared the spot urine collection (using the INTERSALT equation) *v*. the 24DR (without quantifying the discretionary use of table salt) in a large sample of adults in New Zealand, consisting of 3321 participants^(67)^. The authors observed poor agreement between estimated Na intake from spot urine collection and those from 24DR^(67)^. In another study, a plethora of different DM and UM were compared with a PABA-validated 24UCol^(16)^. The assessment of Na intake included an FFQ, a modified 24DR and three equations to convert the spot Na to 24UNa (INTERSALT, Tanaka and Kawasaki). In this study neither DM nor UM provided accurate estimations at individual level, but for group means, the DM and some of the UM may be useful for Na estimation^(19)^. However, the method for the quantification of Na intake has not been clearly described^(19)^.

FFQ are commonly used in dietary Na assessment in population-based studies, having the ability to bypass problems related to day-to-day variability of Na intake and cover larger time periods of intake. The last four decades several FFQ have been designed for the estimation of Na (or salt) intake^(23,24,68–71)^. However, most of them present weak correlations with the 24UCol, ranging from 0·19 to 0·35^(23,24,69,71)^. Furthermore, the available FFQ for Na assessment have been designed for particular ethnic groups^(24,25,69–71)^. To our knowledge, only two of them have been developed for hypertensive subjects^(23,25)^ but until now, there was no FFQ for Na estimation in other high CV risk groups, such as patients with dyslipidaemia, diabetes mellitus, infectious or autoimmune diseases. Recently, McLean *et al.* published a systematic review of the literature, regarding the assessment of dietary Na intake using FFQ and 24UCol^(65)^. This work revealed a poor agreement between estimates of Na from FFQ and 24UCol^(65)^, indicating that the Na FFQ until now are inadequate to estimate the true intake.

The novel NaFFQ was created to accurately estimate Na intake in high CVD risk populations, calculating not only Na derived from food content, but table and cooking Na as well. Our aim was to cover the need of an easily applicable in epidemiological studies and reliable tool for group means of Na intake, which could lead to a better management of high CVD-risk populations. According to our findings, this tool presented the best correlation with – and the lowest bias from – the 24UCol compared with all the existing DM, even when attempts to further improve the accuracy of 24DR were applied. However, despite these promising findings regarding NaFFQ, it provided very wide limits of agreement in Bland–Altman plots, reaching ∼3000 mg/d, indicating that future improvements have to be addressed. A limitation of our study is the use of a single 24UCol. Due to the day-to-day variability in Na intake and excretion, multiple 24UCol are recommended either for assessing accurately usual *individual* Na intake or for a more reliable record of dietary Na in studies investigating its relationship with health or disease^(72,73)^. In our study, our aim was to estimate Na intake in group means and not in individual level, so the use of single 24UCol, which is very common in epidemiological studies, was reasonable. Indeed, the use of a single 24UCol *v*. three to seven 24UCol have been reported to provide similar mean levels of Na excretion at the population level^(74)^. Second, an important limitation to be mentioned is the method used for the quantification of discretionary salt. In our study, the use of dashes of salt, as well as the cut-offs that were designed for processed food, may lead to several concerns and systematic bias. However, until today, the estimation of discretionary salt in studies remains a challenge for the investigators, and there is no generally accepted protocol to be applied in dietary surveys^(75)^. Moreover, the NaFFQ is population specific and has not been externally validated in other populations. Nevertheless, the methodology used here could be used to adapt other FFQ, designed for other population groups, in order to more accurately estimate Na intake.

In conclusion, the available DM and spot urine methods present poor accuracy compared with the gold-standard 24UCol. The new FFQ – specifically designed for Na estimation – is a promising method to detect a mean Na intake at population level in high CVD-risk people. Future validation of this tool in larger populations would verify its accuracy and/or provide evidence for further amelioration, making it a reliable and easy to use clinical tool for Na quantification, in population-based studies. Similar approaches might be useful for other populations.

## Supplementary material

For supplementary material accompanying this paper visit https://doi.org/10.1017/S1368980021004390. click here to view supplementary material
