# Supplementary material for: Dietary sodium estimation methods: accuracy and limitations of old and new methods in individuals at high cardiovascular risk
Source: Public Health Nutr. 2021 Oct 25;25(4):866–78. doi: 10.1017/S1368980021004390 (PMC9991764; doi:10.1017/S1368980021004390)
Supplement: Supplementary file 1 [file S1368980021004390sup001.docx]

**Supplementary Material**

**Supplementary Table 1:** Food Items and questions added in the existing FFQ.

| ***Food items added***  *In cheese food group:*  roquefort, blue cheese, 30 g  parmesan, 30 g  edam, gouda, 30 g  gruyere, 30 g  cream cheese full fat, 30 g  feta cheese and goat cheese, 30 g  mozzarella, 30g  anthotiro (traditional Greek white cheese), 30 g | ***Consumption Frequency***  never/ rarely, 1-3 t/mo, 1-2 t/wk, 3-6 t/wk, 1 t/d, ≥2 t/d  never/ rarely, 1-3 t/mo, 1-2 t/wk, 3-6 t/wk, 1 t/d, ≥2 t/d  never/ rarely, 1-3 t/mo, 1-2 t/wk, 3-6 t/wk, 1 t/d, ≥2 t/d  never/ rarely, 1-3 t/mo, 1-2 t/wk, 3-6 t/wk, 1 t/d, ≥2 t/d  never/ rarely, 1-3 t/mo, 1-2 t/wk, 3-6 t/wk, 1 t/d, ≥2 t/d  never/ rarely, 1-3 t/mo, 1-2 t/wk, 3-6 t/wk, 1 t/d, ≥2 t/d  never/ rarely, 1-3 t/mo, 1-2 t/wk, 3-6 t/wk, 1 t/d, ≥2 t/d  never/ rarely, 1-3 t/mo, 1-2 t/wk, 3-6 t/wk, 1 t/d, ≥2 t/d |
| --- | --- |
| *In cereals and starchy foods food group:*  salty crackers and biscuits, 2 thin pieces (20 g) | never/ rarely, 1-3 t/mo, 1-2 t/wk, 3-6 t/wk, 1 t/d, ≥2 t/d |
| *In meat food group:*  gyros pork, 150 g | never/ rarely, 1-3 t/mo, 1-2 t/wk, 3-6 t/wk, 1 t/d, ≥2 t/d |
| *In fish food group:*  canned or corned fish, 30 g  canned seafood , 30 g | never/ rarely, 1-3 t/mo, 1-2 t/wk, 3-6 t/wk, 1 t/d, ≥2 t/d  never/ rarely, 1-3 t/mo, 1-2 t/wk, 3-6 t/wk, 1 t/d, ≥2 t/d |
| *In nuts and seeds food group:*  salted nuts and seeds, 1 coffee cup (50 g) | never/ rarely, 1-3 t/mo, 1-2 t/wk, 3-6 t/wk, 1 t/d, ≥2 t/d |
| *In margarine food group:*  salted margarine, 1 tsp (5 g) | never/ rarely, 1-3 t/mo, 1-2 t/wk, 3-6 t/wk, 1 t/d, ≥2 t/d |
| *In butter food group:*  salted butter, 1 tsp (5 g) | never/ rarely, 1-3 t/mo, 1-2 t/wk, 3-6 t/wk, 1 t/d, ≥2 t/d |
| *In cooked meals usually prepared with tomato juice*,  refined tomato juice  4 table spoons (60 g) for:  pasta/ pearl barley 1 cup (140 g)  whole meal pasta 1 cup (140 g)  veal (150g)  petit pois (peas) , green beans, okra, artichoke (200 g)  2 table spoons (30 g) for:  pastitsio/ mousakas/ papoutsakia (  Traditional greek dishes), 1 portion(140 g) | *added in the recipe* |
| ***Dietary behaviors related to salt***   1. Which kind of salt do you use? | ----------------------------------------------- |
| 1. How much salt do you use in your cooked meals and salads? | none, a little, moderate amount, much, very much |
| 1. How often do you use table salt in your meals? | never, rarely, often, always |
| 1. Do you believe that salt can cause health problems? | yes, no, I don’t know |
| 1. The amount of salt you eat in your cooked meals is: | I don consume any salt, a little, moderate, a lot |
| 1. The amount of salt you eat in your salads is: | I don consume any salt, a little, moderate, a lot |
| 1. Do you check food labels for salt content? | yes, no |
| 1. Do you buy foods indicating in their package “with less salt”? | yes, no |
| 1. Do you take any measures to control salt intake? | yes, no |
| 1. Do you know if there are recommendations regarding upper limits of daily salt intake? | yes, no, I don’t know |
| g: grams; t: times; mo: month; wk;: week; d: day; tsp: teaspoon | |

**Supplementary Table 2**: Exclusion criteria for incomplete 24h urine collections.

| **Incomplete 24h urine collection if:** |
| --- |
| 1. Urine volume <500mL |
| 1. Urine creatinine <6mmol/day plus 24h urine volume <1000mL or urine creatinine <5mmol/day (1) |
| 1. $\frac{urine creatinine (\frac{mg}{day})}{body weight (kg)}<10.8 or>25.2$ (2) |
| 1. $Males: \frac{urine creatinine (\frac{mg}{day})}{body weight (kg)}<14 or>26$, $Females: \frac{urine creatinine (\frac{mg}{day})}{body weight (kg)}<11 or>20$(3) |
| 1. $Males: \frac{urine creatinine (\frac{mg}{day})}{24 x body weight (kg)}<0.6$, $Females: \frac{urine creatinine (\frac{mg}{day})}{21 x body weight (kg)}<0.6$(4) |

**Supplementary Table 3:** Pearson’s and Spearman correlations & intraclass correlation coefficients between 24h urinary sodium excretion and the other sodium estimation methods in the subgroup of participants after applying the exclusion criteria for incomplete 24h urine collections.

|  |  | **Na estimation methods** |  | **Subgroups**  of sample after applying the exclusion criteria for incomplete 24h urine collections (sensitivity analysis) | | | | |
| --- | --- | --- | --- | --- | --- | --- | --- | --- |
|  |  |  |  | **Exclusion criterion**  **1** | **Exclusion criterion**  **2** | **Exclusion criterion**  **3** | **Exclusion criterion**  **4** | **Exclusion criterion**  **5** |
| ***Spot urine methods*** | | Kawasaki | r | 0.610** | 0.613** | 0.663** | 0.588** | 0.595** |
|  |  |  | ICC (95% CI) | 0.758 (0.603-0.852) | 0.760 (0.604-0.854) | 0.796 (0.641-0.884) | 0.740 (0.584-0.838) | 0.746 (0.507-0.869) |
|  |  |  | *N* | 65 | 64 | 50 | 71 | 37 |
|  |  | Tanaka | r | 0.582** | 0.581** | 0.620** | 0.554** | 0.532** |
|  |  |  | ICC (95% CI) | 0.693 (0.497-0.813) | 0.695 (0.498-0.815) | 0.735 (0.534-0.850) | 0.669 (0.469-0.794) | 0.654 (0.329-0.822) |
|  |  |  | *N* | 65 | 64 | 50 | 71 | 37 |
|  |  | INTERSALT with spot K | r | 0.496** | 0.464** | 0.533** | 0.477** | 0.629** |
|  |  |  | ICC (95% CI) | 0.633 (0.391-0.779) | 0.606 (0.343-0.764) | 0.677 (0.424-0.819) | 0.615 (0.375-0.762) | 0.736 (0.477-0.867) |
|  |  |  | *N* | 62 | 61 | 48 | 68 | 35 |
|  |  | INTERSALT without spot K | r | 0.481** | 0.444** | 0.509** | 0.466** | 0.606** |
|  |  |  | ICC (95% CI) | 0.603 (0.349-0.758) | 0.570 (0.293-0.739) | 0.644 (0.372-0.798) | 0.588 (0.339-0.743) | 0.704 (0.425-0.848) |
|  |  |  | *N* | 65 | 64 | 50 | 71 | 37 |
|  |  | Mage | r | 0.655** | 0.649** | 0.700** | 0.625** | 0.662** |
|  |  |  | ICC (95% CI) | 0.681 (0.476-0.805) | 0.663 (0.445-0.795) | 0.698 (0.468-0.829) | 0.671 (0.473-0.795) | 0.741 (0.498-0.867) |
|  |  |  | *N* | 65 | 64 | 50 | 71 | 37 |
|  |  | Toft | r | 0.577** | 0.580** | 0.670** | 0.554** | 0.695** |
|  |  |  | ICC (95% CI) | 0.680 (0.475-0.805) | 0.680 (0.474-0.806) | 0.757 (0.571-0.862) | 0.659 (0.454-0.788) | 0.787 (0.586-0.890) |
|  |  |  | *N* | 65 | 64 | 50 | 71 | 37 |
| ***Dietary methods*** | Existing dietary methods | 24DR | r | 0.254** | 0.217* | 0.293** | 0.270** | 0.295* |
|  |  |  | ICC (95% CI) | 0.359 (0.062-0.562) | 0.345 (0.034-0.556) | 0.408 (0.099-0.612) | 0.389 (0.112-0.580) | 0.370 (-0.029-0.614) |
|  |  |  | *N* | 108 | 104 | 89 | 112 | 66 |
|  |  | FFQ | r | 0.197 | 0.267* | 0.266* | 0.267* | 0.321* |
|  |  |  | ICC (95% CI) | 0.367 (0.007-0.596) | 0.431 (0.096-0.642) | 0.440 (0.079-0.660) | 0.404 (0.076-0.616) | 0.468 (0.044-0.704) |
|  |  |  | *N* | 78 | 74 | 64 | 82 | 47 |
|  | Improved dietary methods | 24DR+SQ | r | 0.296** | 0.237* | 0.295** | 0.299** | 0.283* |
|  |  |  | ICC (95% CI) | 0.403 (0.126-0.592) | 0.373 (0.075-0.575) | 0.431 (0.134-0.627) | 0.425 (0.164-0.605) | 0.376 (-0.019-0.618) |
|  |  |  | *N* | 108 | 104 | 89 | 112 | 66 |
|  |  | 24DR+15% | r | 0.254** | 0.217* | 0.293** | 0.270** | 0.295* |
|  |  |  | ICC (95% CI) | 0.379 (0.091-0.575) | 0.363 (0.061-0.568) | 0.428 (0.129-0.624) | 0.411 (0.145-0.595) | 0.389 (0.001-0.626) |
|  |  |  | *N* | 108 | 104 | 89 | 112 | 66 |
|  |  | NaFFQ | r | 0.511** | 0.442** | 0.459** | 0.491** | 0.452** |
|  |  |  | ICC (95% CI) | 0.667 (0.429-0.806) | 0.612 (0.331-0.775) | 0.627 (0.334-0.791) | 0.657 (0.423-0.796) | 0.623 (0.260-0.808) |
|  |  |  | *N* | 55 | 54 | 48 | 59 |  |
| * p<0.05; ** p≤0.01. ICC: intraclass correlation coefficient; CI: confidence interval; Na: sodium; 24UNa: 24h urine Na; 24DRNa:24h dietary recalls Na; 24DRNa+15%: 24h dietary recalls Na plus 15% (discretionary Na); 24hDRNa+SQ: 24h dietary recalls Na plus discretionary salt questions; FFQ: food frequency questionnaire.  Exclusion criteria for incomplete 24h urine collections: Exclusion criterion 1: Urine volume <500mL; Exclusion criterion 2: Urine creatinine <6mmol/day plus 24h urine volume <1000mL or urine creatinine <5mmol/day ^(1)^; Exclusion criterion 3: (urine creatinine (mg/day))/(body weight (kg))<10.8 or>25.2 ^(2)^; Exclusion criterion 4: Males: (urine creatinine (mg/day))/(body weight (kg))<14 or>26, Females: (urine creatinine (mg/day))/(body weight (kg))<11 or>20 ^(3)^ Exclusion criterion 5: Males: (urine creatinine (mg/day))/(24 x body weight (kg))<0.6, Females: (urine creatinine (mg/day))/(21 x body weight (kg))<0.6 ^(4)^ | | | | | | | | |

**References**

1. Reinivuo H, Valsta LM, Laatikainen T, Tuomilehto J, Pietinen P. Sodium in the Finnish diet: II trends in dietary sodium intake and comparison between intake and 24-h excretion of sodium. European journal of clinical nutrition. 2006;60(10):1160-7.

2. WHO Regional Office for Europe. Estimation of sodium intake and output: review of methods and recommendations for epidemiological studies. Report on a WHO meeting by the WHO collaborating center for research and training in cardiovascular diseases. Geneva: World Health Organization. 1984.

3. Malekshah AF, Kimiagar M, Saadatian-Elahi M, Pourshams A, Nouraie M, Goglani G, et al. Validity and reliability of a new food frequency questionnaire compared to 24 h recalls and biochemical measurements: pilot phase of Golestan cohort study of esophageal cancer. European journal of clinical nutrition. 2006;60(8):971-7.

4. Joossens JV, J G. Monitoring salt intake of the population: methodological considerations; In Surveillance of the Dietary Habits of the Population with Regard to Cardiovascular Diseases, EURONUT Report 2, pp. 61–73 [GG De Backer, HT Pedoe and P Ducimetiere, editors]. Wageningen: Department of Human Nutrition, Agricultural University. 1984.
